# Profile of melatonin and its receptors and synthesizing enzymes in cumulus–oocyte complexes of the developing sheep antral follicle—a potential estradiol-mediated mechanism

**DOI:** 10.1186/s12958-018-0446-7

**Published:** 2019-01-03

**Authors:** Longfei Xiao, Junjie Hu, Liangli Song, Yong Zhang, Weitao Dong, Yuting Jiang, Quanwei Zhang, Ligang Yuan, Xingxu Zhao

**Affiliations:** College of Veterinary Medicine, Gansu Agricultural University, Lanzhou, 730070 China

**Keywords:** Melatonin, Estradiol, Estrogen receptor, Sheep

## Abstract

**Background:**

Melatonin is an amine hormone that plays an important role in regulating mammalian reproduction. This study aimed to investigate the expression pattern of melatonin synthesis enzymes AANAT and HIOMT and melatonin receptors MT1 and MT2 in sheep cumulus–oocyte complexes (COCs) as well as the change of melatonin level in follicular fluid (FF) during antral follicle development. In this research, we also study the effect of β-estradiol (E2) on MT1 and MT2 expression as well as melatonin synthesis in COCs so as to lay the foundation for further exploration of the regulation mechanism of melatonin synthesis in the ovary.

**Methods:**

COCs and FF were collected from different size (large follicles (diameter ≥ 5 mm), medium follicles (diameter 2–5 mm), and small follicles (diameter ≤ 2 mm)) of antral follicles in sheep ovaries. To assess whether E2 regulates melatonin synthase and its receptors expression in sheep COCs and whether it is mediated through estrogen receptor (ER) pathway. The collected COCs were cultured in vitro for 24 h and then treat with 1 μM E2 and/or 1 μM ICI182780 (non-selective ER antagonist). The expression of AANAT, HIOMT, MT1 and MT2 mRNA and protein were determined by qRT-PCR and western blot. The melatonin level was determined by ELISA.

**Results:**

The expression of AANAT, HIOMT, MT1 and MT2 were significantly higher expression in the COCs of small follicles than in those of large follicles (*P* < 0.05). However, the melatonin level was significantly higher in large follicle FF than in small follicle FF (*P* < 0.05). Further, the expression of AANAT, HIOMT, MT1, and MT2 and melatonin production were decreased by E2 treatment (*P* < 0.05), but when ICI182780 was added, the expression of AANAT, HIOMT, MT1, and MT2 and melatonin production recovered (*P* < 0.05).

**Conclusions:**

We suggest that sheep COCs can synthesize melatonin, but this ability is decreased with increasing follicle diameter. Furthermore, E2 play an important role in regulated the expression of MT1 and MT2 as well as melatonin synthesis in sheep COCs through the ER pathway.

## Background

Melatonin (*N*-acetyl-5-methoxytryptamine) is an indoleamine originally identified in the pineal gland, where it is synthesized enzymatically from serotonin (5-hydroxytryptamine) by the sequential action of arylalkylamine *N*-acetyltransferase (AANAT) and hydroxyindole-*O*-methyltransferase (HIOMT) [1–3]. Both AANAT and HIOMT have been considered rate-limiting steps in melatonin production. Generally considered, the synthesis of melatonin by the pineal gland is regulated by external photoperiodic cues. However, estrogen, an important ovarian hormone, also influences melatonin synthesis. Study have shown that a higher dose of estradiol benzoate reduces the activities of AANAT and HIOMT in the pineal gland of ovariectomized rats, and causes a decrease in melatonin concentration in peripheral blood [4].

The pineal gland is thought to be the main site of melatonin synthesis; however, many extrapineal tissues, such as the retina [5], gastrointestinal tract [6], spleen, liver, kidney, heart [7],testes [8], and ovaries [9] secrete melatonin. AANAT and HIOMT have been found in the ovaries of rats and humans [9–12]. The affinities of AANAT and HIOMT for their substrates in the ovaries are approximately equal to those in the pineal glands, which indicate that the ovary can also synthesize melatonin [11, 12]. Moreover, the granulosa cells, including those forming the cumulus oophorus [13, 14], and the oocytes [15] have been reported to detect the melatonin-synthesizing enzymes, and studies have found that human [16] and bovine [13] cumulus–oocyte complexes (COCs) can also synthesize melatonin. Rather, these cells use the melatonin they produce for their own benefit or for that of their neighboring cells as an antioxidant and as an autocrine or paracrine agent [17].

However, high levels of steroid hormones and metabolic demands characterize the developing follicle, and as a result oxidative stress may ensue [18–20], thereby affecting the development of follicles and oocytes [21]. Melatonin, a powerful antioxidant, has the ability to scavenge reactive oxygen species (ROS) and reactive nitrogen species (RNS) via receptor-independent actions [22–24]. Melatonin not only directly protects the COCs from oxidative damage, but also promotes the COCs to secrete antioxidant proteins such as CuZn-SOD, Mn-SOD, and glutathione peroxidase GPx to protect itself [25]. In addition to its antioxidant properties, melatonin has also been identified to have other functions in oocyte and follicular development. So far, melatonin has been found in the human follicular fluids, and its concentrations increase with follicular development [12]. The two melatonin high-affinity membrane receptors, MT1 and MT2, have been detected in granulosa cells, cumulus cells, and oocytes of human [26] and rat [27], indicating that melatonin is involved in regulation of steroidogenesis modulation [28, 29], follicular development [30–32], oocyte maturation [33–36], ovulation [37] and luteinization [38] via its receptor pathway.

However, most of the research on melatonin and its related proteins in follicular development have focused on humans and rodents, and there are few reports in sheep. Therefore, in this study, sheep were used as the experimental animals. Immunohistochemistry, real-time PCR, and western blotting were used to detect whether AANAT, HIOMT, MT1, and MT2 are expressed in sheep COCs and to analyze the expression of AANAT, HIOMT, MT1, and MT2 mRNA and protein in COCs of follicles of different sizes in sheep. We also assessed the melatonin levels in follicular fluid (FF) from follicles of different sizes. In addition, we added exogenous E2 and estrogen receptor inhibitor ICI182780 in vitro to test whether E2 regulates melatonin synthase and its receptor expression in sheep COCs.

## Methods

### Materials and sample collection

Cell culture medium (TCM199) was purchased from GIBCO (GIBCO, CA, USA). ICI182780 (ab120131) and Rabbit polyclonal Anti-MT2 antibody (ab203346) were purchased from Abcam (Abcam, UK). Rabbit polyclonal Anti-MT1 antibody (PL-0300967) was purchased from PL (PL, CAM). Rabbit polyclonal Anti-HIOMT antibody (orb2459) was purchased from Biorbyt (Biorbyt, UK). β-Estradiol (E2) (50-28-2) and Bovine Serum Albumin (BSA) was purchased from Sigma–Aldrich (St. Louis, Missouri). Rabbit polyclonal Anti-AANAT antibody (bs-3914R) and Rabbit polyclonal Anti-β-actin antibody (bs-0061R) were purchased from Bioss (Bioss, Beijing, China).

In this experiment, ovaries were obtained from adult sheep (body weight: 35–55 kg) killed at Lanzhou slaughterhouse. Ovaries samples were held in Dulbecco’s PBS (DPBS; Ca^2+^- and Mg^2+^-free) at 30–35 °C containing streptomycin (100 IU/mL) and penicillin (50 mg/mL) and send to the laboratory within 3 h. All experimental procedures involving animals were approved by the institutional animal care and local ethics committee.

### COCs collection

Total 128 sheep ovaries were used in this experiment. Six ovaries were randomly selected and fixed in 4% formaldehyde for 24 h, then embedded in paraffin for immunohistochemistry. Sheep ovary COCs and FF were aspirated from large follicles (diameter ≥ 5 mm), medium follicles (diameter 2–5 mm), and small follicles (diameter ≤ 2 mm) from 60 sheep ovaries. Then, the collected FF was clarified by centrifugation for 10 min at 3000 rpm. The supernatant fluids were passed through a 0.45-mm filter and stored at − 80 °C until analysis of the melatonin concentration. The sheep ovary COCs were washed three times in DPBS and stored at − 80 °C until analysis of AANAT, HIOMT, MT1, and MT2 mRNA and protein expression.

### Cell culture and treatment

The COCs for in vitro cultured were aspirated from follicles by cutting the surface of 1–6 mm follicles from the remaining ovaries. Only COCs with fine homogenous granular cytoplasm surrounded by compact layers of granulosa cells were selected for maturation. After washing three times with DPBS and one time with TCM199, the COCs chosen for the experiment were placed in a four-well plate, each well containing 50 COCs in 700 μL of TCM199 with 5 mg/ml BSA. COCs were cultured in a humidified incubator (5% CO2) at 38.5 °C in order to separate from the hormone environment in vivo. After 24 h cultured, the cell culture medium were changed with fresh medium which added with 5-HT (melatonin precursor 5-hydroxytryptamine) to a final concentration of 1 μM [15]. Then, the COCs were treated with: (1) only 0.1% DMSO (*w*/*v*), as a Control group; (2) 1 μM E2; (3) 1 μM ICI182780 (non-selective ER antagonist); (4) 1 μM E2 and 1 μM ICI182780 + E2 for 24 h. Then, the COCs and culture medium were collected and stored at − 80 °C until analysis of AANAT, HIOMT, MT1 and MT2 mRNA and protein expression and melatonin production.

### Total RNA isolation and real-time–polymerase chain reaction (qRT-PCR)

Total RNA was extracted using Trlquick Reagent (Solarbio, Beijing, China). RNA purity and integrity were determined as described previously [39]. Then, the RNA was reverse-transcribed to single-stranded cDNA with a reverse transcription kit (Promega, Wisconsin, America) for qRT-PCR.

The qRT-PCR primers were designed according to the *Ovis aries AANAT*, *HIOMT*, *MT1*, *MT2*, and *β-actin* gene sequences showed in Table 1. qRT-PCR was conducted with an FTC-3000 thermocycler (Funglyn Biotech, Canada) at a 20-μl reaction volume consisting of 1 μl of cDNA, 1 μl of forward primer, 1 μl of reverse primer, 10 μl of 2× SYBR Green II PCR mix (TaKaRa, Shiga, Japan), and 7 μl of nuclease-free H_2_O. The PCR conditions were as follows: 95 °C for 30 s, 95 °C for 5 s, and 60 °C for 30 s for a total of 40 cycles; 95 °C for 30 s; 60 °C for 90s; and 95 °C for 10 s. Four replicates were set for each sample to ensure the accuracy of the relative expression of the target gene in the samples. The 2^-△△Ct^ method was used to determine the expression of *AANAT*, *HIOMT*, *MT1*, and *MT2* mRNA relative to *β-actin* according to the system-generated Ct value [40].

**Table 1:** Primers used in real-time RT-PCR

Genes	Primer sequences (5′-3′)	Length (bp)	Accession No.
*HIOMT*	F: GCTGTACTCGCTGAACATGC	141	NM_001306120.1
	R: CTGCCCAAGACTGCATCGTA		
*AANAT*	F: CCCCCTGAATCTGGACGAG	108	NM_001009461.1
	R: CACAGGGAGCCGATGATGAAGG		
*MT1*	F: GGTGTTCCATTTCATAGTTCC	169	NM_001009725.1
	R: GGCAAAGAGGACAAAAACC		
*MT2*	F: AGGTCAAGGCGGAGAGC	148	NM_001130938.1
	R: GCCACTTCTTCGGGGTCAA		
*β-actin*	F: CTTCCAGCCTTCCTTCCTGG	180	NM_001009784.2
	R: GCCAGGGCAGTGATCTCTTT		

### Protein extraction and western blotting

The COCs were homogenized and lysed in RIPA buffer (Solarbio) containing aprotinin, sodium orthovanadate, phenyl methylsulfonyl fluoride (PMSF; Solarbio), and protease inhibitor cocktail for 30 min, followed by centrifugation at 12,000 rpm and 4 °C for 15 min. Protein levels were measured with the enhanced BCA protein assay kit (Bio-Tek, VT, USA). Then, 4× sodium dodecyl sulfate (SDS) loading buffer (Solarbio) was added, and proteins were denatured at 98 °C for 10 min. Detailed information on western blotting has been given in earlier papers [41]. The antibody concentrations of AANAT, HIOMT, MT1, MT2 and β-actin were 1:500, 1:1000, 1:1000, 1:300 and 1:1000 respectively.

### Melatonin levels in FF from follicles of different sizes and in COC culture medium

The melatonin levels in FF from follicles of different sizes and COC culture medium were quantified using an enzyme-linked immunosorbent assay (ELISA; sheep melatonin ELISA kit; USCN, Wuhan, China). In brief, cell culture medium was collected and centrifuged at 3000 rpm for 15 min at 4 °C; the resulting supernatant was extracted and stored at − 80 °C. Then, 10 μl of the extracted proteins from the cell culture medium were added to 96-well plates and 40 μl of the diluted samples were added to each pre-coated well. After incubation for 1 h at 37 °C with shaking and five washes, 100 μl of detection antibody-horseradish peroxidase (HRP) was added to each well, and the plate was incubated for 1 h at 37 °C. After shaking and five washes, 100 μl of substrate solution was added and the samples were incubated for 15 min at room temperature in the dark. The reaction was stopped by adding 50 μl of stop solution, and the absorbance was measured immediately at 450 nm against a standard curve. Each sample was tested in duplicate and the net absorbance was obtained when the absorbance of the negative control (blanks, without sample) was subtracted from that of the samples. The melatonin level in cell culture medium was expressed as pg/ml.

### Immunohistochemical staining

Expression of AANAT, HIOMT, MT1, and MT2 was analyzed by immunocytochemistry. Briefly, ovary samples were fixed using 4% paraformaldehyde (*w*/*v*) in 0.1 M phosphate buffer (pH 7.4) and embedded in paraffin. Sections (4 μm) were mounted onto gelatin/poly-l-lysine–coated glass slides. The sections were dried on the slides in a 60 °C incubator for 2 h. They were dewaxed twice in xylene for 15 min each and rehydrated through graded ethanol solutions (100, 90, and 70%, (*v*/v)). Then, the sections were dewaxed in water, washed three times with 0.01 M PBS (pH 7.4) for 3 min each, incubated with 0.3% H_2_O_2_ (*w*/*v*) for 10 min to block endogenous peroxidase activity, and then stained using the immunohistochemical SP procedure. Detailed information on SP has been given in earlier papers [41]. The antibody concentrations of AANAT, HIOMT, MT1, and MT2 were 1:100, 1:200, 1:100, and 1:200 respectively. Color development reaction was achieved using diaminobenzidine (DAB), and nuclear counterstaining was performed with hematoxylin. The negative control was incubated with PBS instead of the primary antibody, and the remaining conditions and steps were the same. The images were observed and photographed using an Olympus-DP71 optical microscope (Olympus, Japan).

### Statistical analysis

Statistical analyses were performed using SPSS version 10.0 (IBM Corporation, NY, USA). All data were tested for normality and homoscedasticity, then subjected to a one-way ANOVA, followed by Duncan’s multiple test to determine differences. All quantitative data are presented as mean ± SEM. *P* < 0.05 was considered significant.

## Results

### Immunohistochemical staining of AANAT, HIOMT, MT1, and MT2 in sheep COCs

**Fig. 1:**
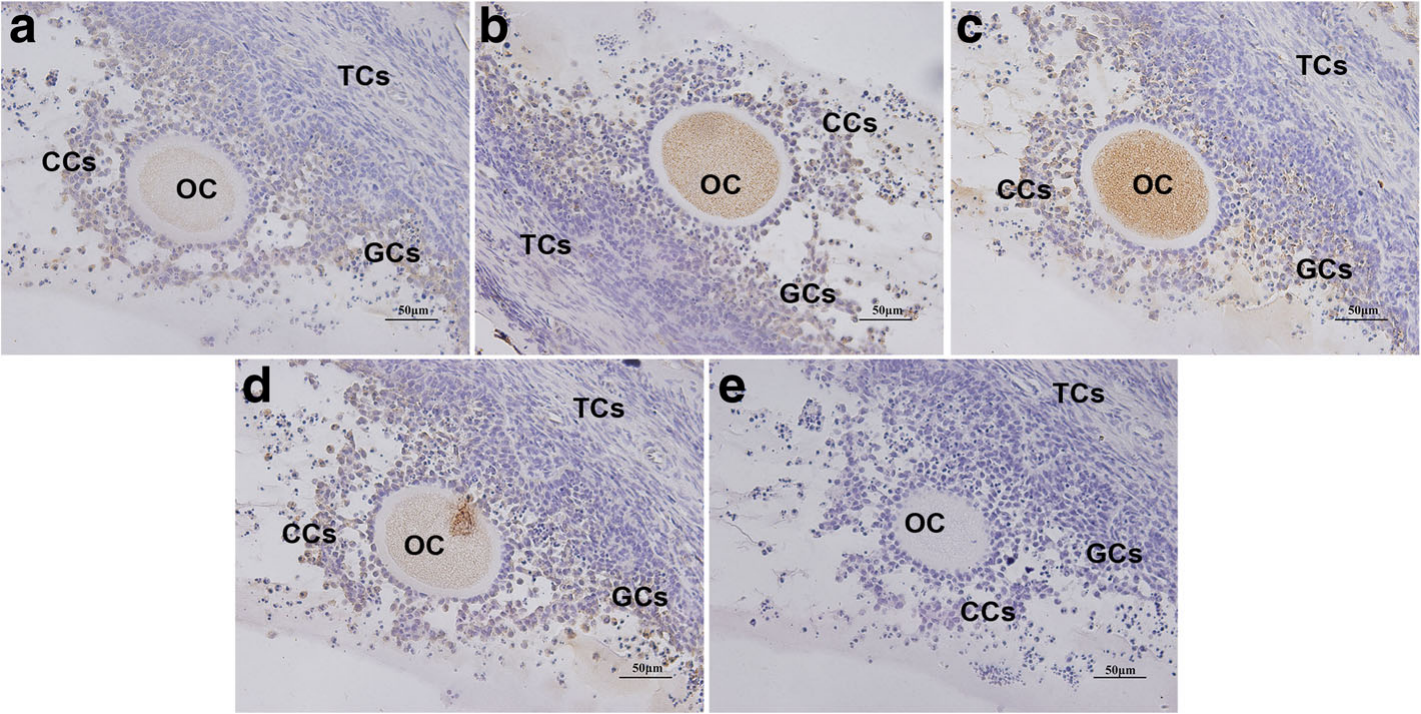
Immunohistochemical localization of AANAT,HIOMT, MT1, and MT2 in sheep ovaries. **a** AANAT, **b** HIOMT, **c** MT1, **d** MT2, **e** negative control. OC: oocyte; GCs: granulosa cells; CCs: cumulus cells: TCs: theca cells. Scale bars correspond to 50 μm

The localization of AANAT, HIOMT, MT1, and MT2 in COCs in sheep ovaries was analyzed using immunohistochemical staining. As shown in Fig. 1, AANAT, HIOMT, MT1, and MT2 were found to be expressed in the same parts, prominently localized to the cumulus cells and oocytes, but with almost no expression in thecal cells.

### Quantification of melatonin levels in FF of follicles of different sizes

The melatonin levels in FF from follicles of different sizes in sheep were detected by ELISA. The results are shown in Table 2. The lowest melatonin level was seen in the small follicles (19.54 ± 1.64 pg/ml, *P* < 0.05), but there was no significant difference between medium follicles and large follicles (respectively, 26.12 ± 2.88 pg/ml and 29.83 ± 3.29 pg/ml, *P* > 0.05). The analysis showed that the melatonin level in the small FF was significantly lower than that in the medium FF and the large FF (*P* < 0.05).

**Table 2:** Melatonin levels in small, medium, and large follicles

Follicle size	Number of follicles	Melatonin (pg/ml)
Large follicles	38	29.83 ± 3.29^a^
Medium follicles	47	26.12 ± 2.88^a^
Small follicles	102	19.54 ± 1.64^b^

Note: FF was collected from 60 sheep ovaries. Data are the mean ± SEM value. Different letters represent a significant difference in the same group (*P* < 0.05)

### AANAT, HIOMT, MT1, and MT2 mRNA and protein expression in sheep COCs from follicles of different sizes

**Fig. 2:**
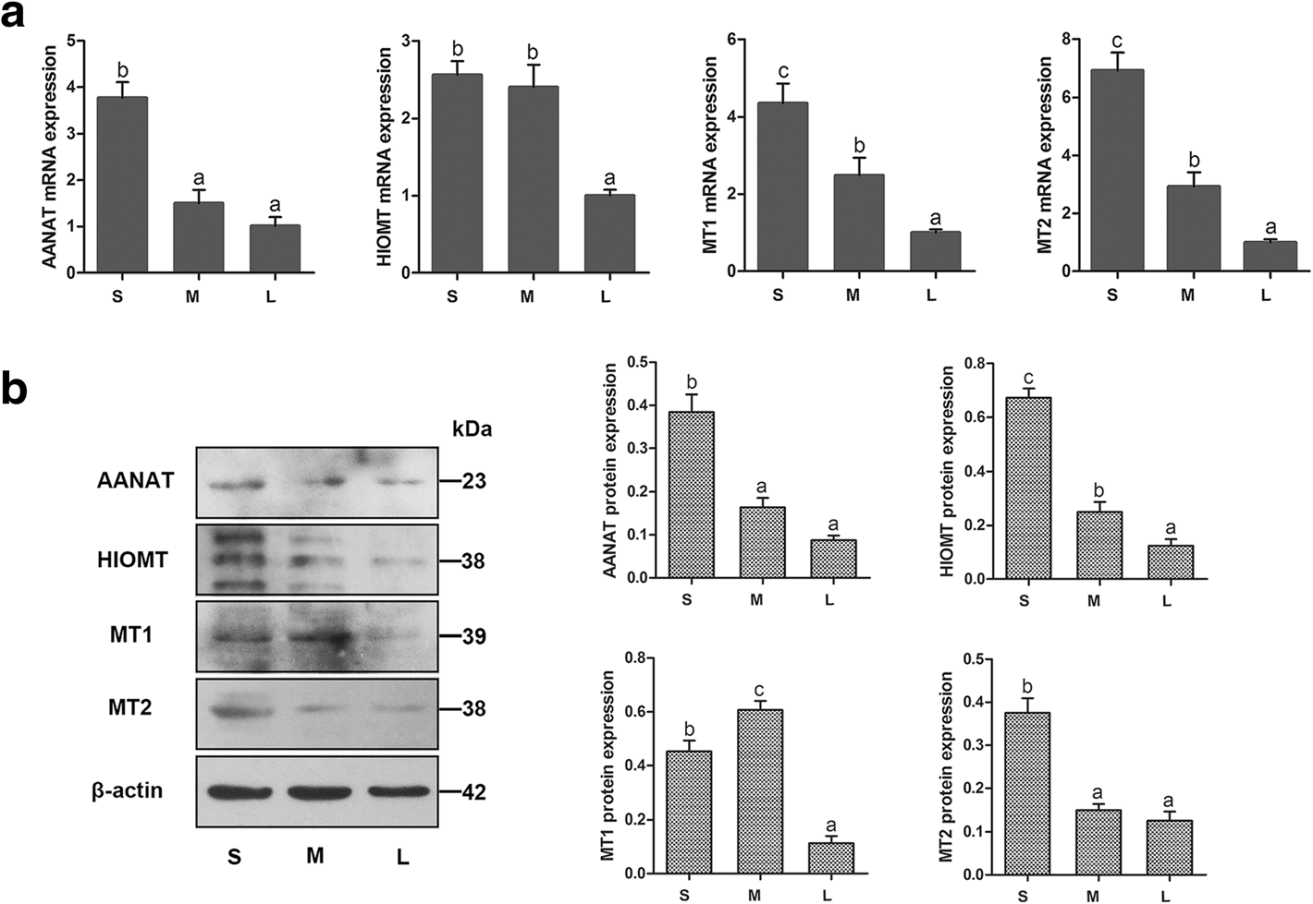
Relative expression of AANAT, HIOMT, MT1, and MT2 in COCs of follicles of different sizes. **a** The relative expression of *AANAT*, *HIOMT*, *MT1*, and *MT2* mRNA. Independent experiments were performed three times, with *β-actin* as an internal control. **b** Western blotting of AANAT, HIOMT, MT1, MT2 and the relative expression of AANAT, HIOMT, MT1, and MT2 protein in COCs of follicles of different sizes. β-actin was used as an internal control. Data are means ± SEM. Different superscript letters (a-c) indicate statistically significant differences (*P* < 0.05). S: small follicle; M: medium follicle; L: large follicle

The mRNA and protein expression levels of AANAT, HIOMT, MT1, and MT2 in sheep COCs from antral follicles of different sizes are shown in Fig. 2. *AANAT*, *HIOMT*, *MT1*, and *MT2* mRNA expression levels in COCs were decreased with increasing follicle diameter, and the expression levels in small follicle COCs were significantly higher than in large follicle COCs (*P* < 0.05, Fig. 2a); however, there was no significant difference in *AANAT* levels between medium follicles and large follicles, and there was no significant difference in *HIOMT* levels between small follicles and medium follicles. A similar trend was observed for protein expression (Fig. 2b); these proteins were significantly decreased in large follicles compared with small follicles (*P* < 0.05); however, the highest expression level of MT1 protein was seen in medium follicles (*P* < 0.05), and there was no significant difference in MT2 protein levels between medium follicles and large follicles.

### Effect of E2 and ER antagonist ICI182780 on melatonin production and the expression of AANAT, HIOMT, MT1, and MT2 mRNA and protein in sheep COCs

**Fig. 3:**
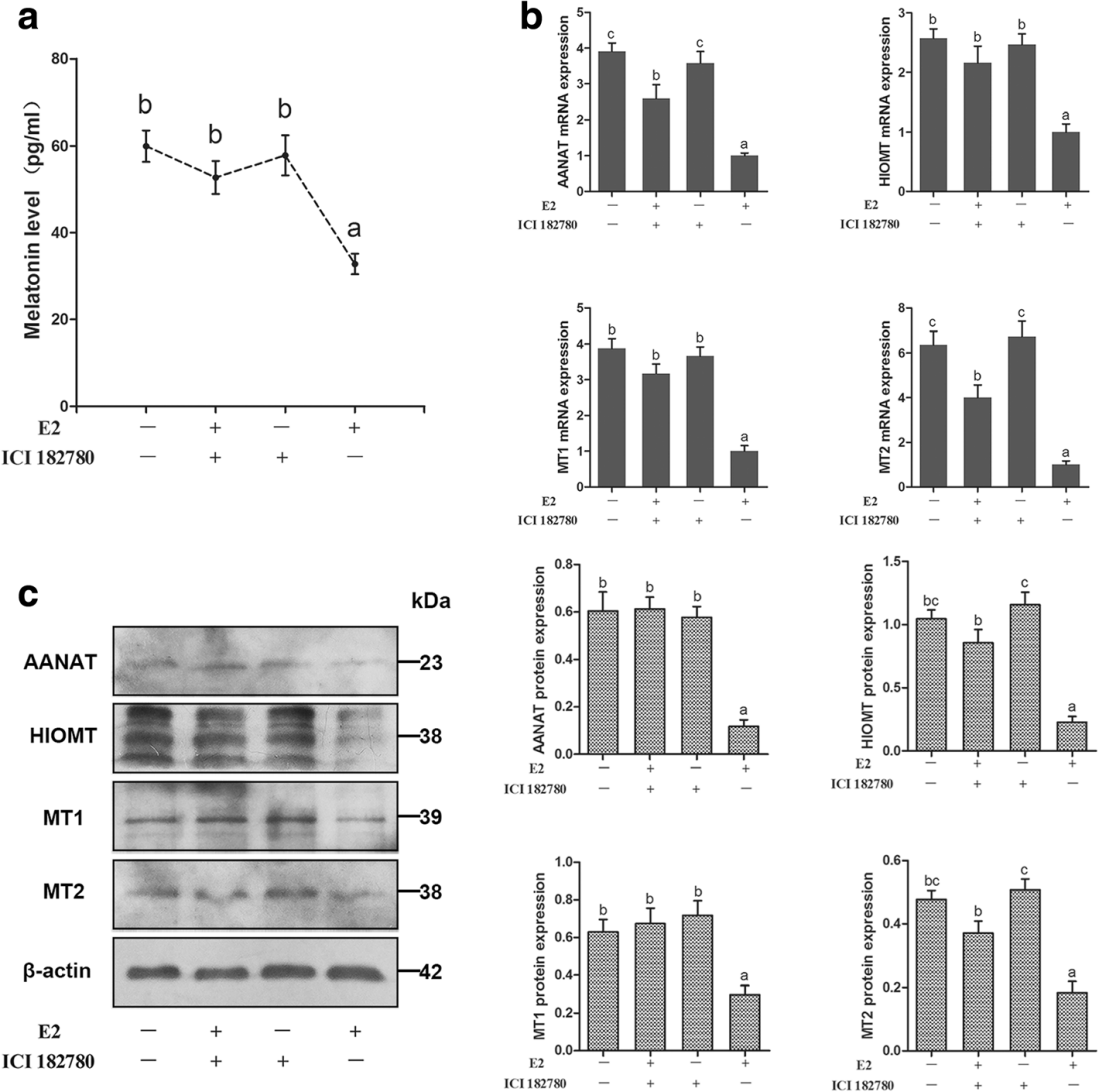
Effect of E2 and ER antagonist ICI182780 on melatonin production and the expression of AANAT, HIOMT, MT1, and MT2 mRNA and protein in sheep COCs. **a** Melatonin levels (*n* = 6 per group). **b** The relative expression of *AANAT*, *HIOMT*, *MT1*, *and MT2* mRNA. Independent experiments were performed three times, with *β-actin* as an internal control. **c** Western blotting of AANAT, HIOMT, MT1, MT2 and the relative expression of AANAT, HIOMT, MT1, and MT2 protein in each COCs treatment group. β-actin was used as an internal control. Different superscript letters (a-c) indicate statistically significant differences (*P* < 0.05)

To examine whether melatonin production was affected by treatment of COCs with 1 μM E2, 1 μM ICI182780, or a combination of 1 μM E2 and 1 μM ICI182780, the cell culture medium was collected and the melatonin concentration was examined. As shown in Fig. 3a, E2 significantly decreased the melatonin level (*P* < 0.05). ICI182780 had no effect on melatonin production, but when COCs were co-cultured with E2 and ICI182780, the melatonin production was significantly increased compared with that by E2 treatment (*P* < 0.05).

Moreover, E2 significantly decreased the AANAT and HIOMT mRNA and protein expression in COCs (*P* < 0.05, Fig. 3b and c), but ICI182780 alone had no effect on the expression of AANAT and HIOMT mRNA and protein in COCs. However, the combination of E2 and ICI182780 significantly increased the AANAT and HIOMT mRNA and protein expression in COCs compared with the E2 group (*P* < 0.05). The MT1 and MT2 mRNA and protein expression levels were similar to those of AANAT and HIOMT (Fig. 3b and c).

## Discussion

AANAT and HIOMT are the key enzymes in the synthesis of melatonin, and also key to determine whether tissues or cells synthesize melatonin. MT1 and MT2 are the main receptors of melatonin, exerting biological activity. In this study, we not only detected the expression of MT1 and MT2, but also that of AANAT and HIOMT proteins in sheep COCs. This finding is consistent with the known presence of AANAT and HIOMT in human [16] and bovine [13] COCs and MT1 and MT2 in goat [42], yak [43], and human COCs [26]. This indicates that the COCs of sheep not only contain the melatonin synthetic pathway but are also a target of melatonin function.

In this study, we found that melatonin exists in sheep FF, and that the melatonin level increased with increasing follicle diameter. This finding is similar to findings in humans [12]. The melatonin in the follicle originates not only from self-secretion by the cells in the follicle, but also from enrichment in the blood [11, 12, 44]. Although the concentration of melatonin increases with the diameter of the follicle, the ability of the sheep COC itself to synthesize melatonin as the follicle develops is still unclear. Thus, we investigated the expression patterns of AANAT and HIOMT mRNA and protein in sheep COCs in follicles of different sizes. The result showed that the expression of AANAT and HIOMT mRNA and protein in sheep COCs in follicles of different sizes varies, and that the expression of AANAT and HIOMT in small follicle COCs is significantly higher than that in large follicle COCs. These findings suggest that although the melatonin level increases with the increase in follicle diameter, the ability of the COC itself to synthesize melatonin is decreased. Studies showed that with the development of the antral follicle, the amount of vascular tissue is increased; hence, substance exchange between FF and blood is increased [45, 46]. Another study showed that the ovarian cells do not discharge melatonin into the general circulation [17]. These findings suggest that the melatonin in large follicles mainly originated from blood. A study in rat ovaries showed that AANAT levels in oocytes increased progressively from primordial follicles to antral follicles [15]. This suggests that in preantral follicles, the follicle cells themselves synthesize melatonin and may be the main source of melatonin in follicle.

We also investigated the expression patterns of MT1 and MT2 mRNA and protein in sheep COCs in follicles of different sizes in this study. The result showed that the expression patterns of MT1 and MT2 in sheep COCs in follicles of different sizes are similar to the expression patterns of AANAT and HIOMT, and that the expression of MT1 and MT2 in small follicle COCs is significant higher than in large follicle COCs. One study showed that estrogen exposure not only downregulates MT1 in rat ovaries [47], but also reduces the activity of AANAT and HIOMT in the pineal gland of ovariectomized rats, and causes a decrease in melatonin concentration in peripheral blood [4]. E2, an ovarian hormone, plays an important role in the development of follicles and oocytes [48, 49]. In addition, E2 levels in the dominant follicles are significantly higher than those in the atretic follicles and peak before ovulation [50]. Sheep large FF contain much higher levels of E2 up to 1 μM [51]. Thus, we speculate that the drastic decrease in melatonin-related proteins in sheep COCs of large follicles is associated with the high level of E2 in these follicles.

In order to test this hypothesis, we simulated the high concentration of E2 in the large follicles in vitro by treating the COCs with 1 μM E2 after 24 h of culture. The results showed that 1 μM E2 significantly reduced the expression levels of MT1, MT2, AANAT, and HIOMT in COCs and decreased the melatonin level in the culture medium. This indicated that the high level of E2 in large follicles not only inhibits the expression of melatonin membrane receptors MT1 and MT2 in the COCs, but also inhibits the expression of melatonin synthesis enzymes AANAT and HIOMT, and thus inhibits melatonin production. However, when E2 and ICI182780 were both added to the cultured COCs, the expression levels of MT1, MT2, AANAT, and HIOMT and melatonin production were restored. Thus far, there are few reports about how E2, through the estrogen receptor (ER), regulates the expression of melatonin synthase and melatonin receptor and the synthesis of melatonin. One study showed that the MT1 receptor is upregulated in estrogen receptor-negative cells (MDA-MB-231) and downregulated in estrogen receptor positive cells (MCF-7) [52]. Our results show that E2 inhibits the expression of melatonin synthesis enzymes AANAT and HIOMT through ER, thus inhibiting the production of melatonin in COCs, whereas the expression of melatonin receptors MT1 and MT2 in COCs is also inhibited by E2 through ER.

Many studies have reported the effects of melatonin on inhibition of the synthesis and function of estrogen. Chuffa et al. [53] showed that the regulation of estrogen secretion by melatonin in the body mainly through the neuroendocrine-gonad axis affects ovarian function and downregulates the secretion of estrogen in the ovary. Melatonin also can act as a selective estrogen receptor modulator (SERM) by reducing the amount of estrogen binding to ER receptors and inhibiting the binding of the E2-ER complex to the DNA [54]. Recently, melatonin has been shown to regulate the activity of some enzymes (aromatase, sulfatase, 17ß-hydroxysteroid dehydrogenase, and estrogen sulfotransferase) responsible for the local synthesis of estrogens in cultured human breast cancer cells, thus behaving as a selective estrogen enzyme modulator (SEEM) [55–57]. However, there are few reports about the effects of estrogen on inhibiting the synthesis of melatonin and expression of its receptors. This experiment for the first time demonstrated the inhibitory effect of E2 on melatonin production and expression of related proteins in sheep COCs.

This study also has several limitations. The ER inhibitor ICI182780 is widely used in basic research and has been proven to effectively inhibit ER function [58]. However, classic ER is made up of two forms, ER-α and ER-β [59, 60], and both are expressed in sheep follicle [61, 62]. In this study, because we primarily focused on the effects of E2, we did not investigate which ER, either ER-α or ER-β, participates in this regulatory process. This question can be answered in our future experiments by using certain specific commercial ligands of ER-α and ER-β.

## Conclusions

Our results draw the conclusion that sheep COCs can synthesize melatonin and are also a target of melatonin action. E2 reduces the expression of MT1 and MT2 and inhibits the expression of AANAT and HIOMT in sheep COCs and ultimately inhibits COC synthesis of melatonin through ER. This study provides a theoretical basis for further study on the synthesis of melatonin in COCs by E2 regulation.

## Abbreviations

AANAT: Arylalkylamine N-acetyltransferase
BSA: Bovine serum albumin
COCs: Cumulus–oocyte complexes
E2: β-estradiol
FF: Follicular fluid
HIOMT: Hydroxyindole-O-methyltransferase
MT1: Melatonin receptor type 1A
MT2: Melatonin receptor type 1B
